# Persistent (Anxiety and Depression) Affected Academic Achievement and Absenteeism in Nursing Students

**DOI:** 10.2174/1874434601812010171

**Published:** 2018-08-31

**Authors:** Mohannad Eid Abu Ruz, Hekmat Yousef Al-Akash, Samiha Jarrah

**Affiliations:** 1 College of Nursing, Applied Science Private University, Amman, Jordan; 2 Clinical Nursing Department, Applied Science Private University, Amman, Jordan; 3 Faculty of Nursing, Applied Science Private University, Amman, Jordan

**Keywords:** Anxiety, Depression, Academic achievement, Absenteeism, Collegial life, Education

## Abstract

**Background:**

Anxiety and depression are common among nursing students due to different factors. When they are minimal and not persistent, they work as stimuli for good achievement. However, when they are high or persistent they have negative consequences (*i.e.* low academic achievement and higher absenteeism rates).

**Objective:**

The purpose of this study was to check the effect of persistent anxiety and depression on nursing student academic achievement and absenteeism rate.

**Methods:**

A prospective observational correlational design with a convenience sample of 170 students enrolled in the undergraduate and graduate programs-college of nursing at a private university in Amman, Jordan. Anxiety and depression were measured twice at the beginning of the semester and then two months later by Hospital Anxiety and Depression Scale. Data regarding grade point average, number of absenteeism and the gender of the students; were collected from the electronic system of the university.

**Results:**

Persistently anxious group has lower grade point average than persistently non-anxious group (mean [SD], 64.1 [13.8] *vs*. 73.1 [12.3], P< .001). Moreover, they have higher absenteeism rate than persistently non-anxious group (7.62 [5.7] *vs*. 4.0 [3.4], P< .001) and higher than transiently anxious group (7.62 [5.7] *vs*. 4.7 [4.6], P< .05). Persistently depressed group has lower grade point average than persistently non-depressed group (64.0 [13.8] *vs*. 73.2 [13.0], P< .001) and lower than transiently depressed (64.0 [13.8] *vs*. 71.7 [10.6], P< .01).

**Conclusion:**

Nursing administrators should search for the underlying causes for these negative emotions. Furthermore, setting strategies to control these negative emotions is highly recommended.

## 1 INTRODUCTION

All over the world, nursing students are exposed to multiple challenges during their collegial life [1]. One of these challenges is vulnerability to emotional instability that may include high levels of anxiety [2-8], and depressive symptoms [1, 4, 5, 9-11]. However, there is a universal agreement that nursing is a stressful profession and nursing students along with other health care professionals are more likely to experience higher levels of anxiety, stress, and depression than students in other fields [7, 12-15].

Specialized literature indicated that nursing students identified multiple and various sources for their anxiety and depression. Some of these sources were considered unique to nursing students such as courses’ structure, clinical experiences, and lack of faculty support [16, 17], efforts to meet the academic requirements [13, 14, 17-19], hours of work, the nature of the profession such as working with sick persons, and fear of failing their exams [20]. In addition to that, the nature of the nursing program curriculum is considered as a challenging educational experience for most students. The life and death events, and lack of course schedule flexibility places nursing students in susceptible psychological positions.

Students’ academic success was linked to their emotional stability [12]. The experienced negative emotional reactions may impact nursing student’ academic performance as demonstrated by lower Grade Point Average (GPA) [21-23] and student’s active attendance to classes. Lower GPAs among nursing students was associated with negative emotional reactions such as anxiety and depression [4, 7]. When nursing students’ experienced high levels of stress, it led to more anxiety, anger, and depression. Literature in this field highlighted the consequences of the increased levels of anxiety and depression [24].

This experienced anxiety may also affect their ability to properly prepare for the examination to achieve satisfactory grades in their exam that may lead to low GPA achievements [25]. On the other hand, low GPA achievements may lead to further anxiety, anger, and depression [24], discouragement, absenteeism, and even withdrawal from the college and/or from the profession, impaired role transition, burnout, and poor job performance [26-29].

Although research indicated that anxiety and depression exist among nursing students, only minimal research available to clarify that if these two negative emotional impacts are transient (on entry/admission to the college) or persistent (continues over the period of collegial life) [30]. In Jordan, no research exists linking these psychological conditions to GPA and absenteeism at two/several points of their collegial life, at enrollment/Fentry and during study period. Therefore, the purpose of this study was to identify the effects of persistent (depression and anxiety) on the GPA and absenteeism among nursing students. Identification of anxiety and depression levels and their impact among nursing students may help faculty members and education leaders in setting strategies to help students overcome these negative emotional consequences, achieve more satisfactory GPA, and report less absenteeism in their classes. Consequently, higher commitment to nursing, lower withdrawal from the profession, and better patients’ care.

### 1.1 Research Hypothesis

Hypothesis 1: Participants who have high anxiety scores at both time points (persistently anxious) will have lower GPA than participants who have high anxiety scores at only one-time point (transiently anxious) or participants who have low anxiety scores at both time points (persistently non-anxious). Hypothesis 2: Participants who are persistently anxious will have higher absenteeism rate than participants who are transiently anxious or participants who are persistently non-anxious. Hypothesis 3: Participants who are persistently depressed will have lower GPA than participants who are transiently depressed or participants who are persistently non-depressed. Hypothesis 4: Patients who are persistently depressed will have higher absenteeism rate than participants who are transiently depressed or participants who are persistently non-depressed.

## 2 MATERIALS AND METHODS

### 2.1 Design, Sample, and Setting

A prospective observational correlational design was used in this study. A convenient sample of *all* students enrolled in the undergraduate and graduate programs-college of nursing at a private university in Amman, Jordan and agreed to participate was included in the study. The total number of the students enrolled in this college was 300 students; 250 baccalaureate students and 50 master students. The sample consisted of 130 baccalaureate students and 40 master students; response rate 68.0%. Students who have a confirmed diagnosis of anxiety and depression were excluded from the study. To make sure that this sample size was sufficient to get statistical significant difference, power analysis was done using the G*power software. Statistical test was ANOVA with 3 groups, type 1 error 0.05, and medium effect size of 0.25, and power of 0.8. Based on these assumptions, the needed sample size was 159 participants. Therefore, 170 participants were considered enough for the purposes of the analyses.

### 2.2 Measurement of Variables

#### 2.2.1 Anxiety And Depression

 Anxiety and depression were defined as the total score of the anxiety/depression subscales of Hospital Anxiety and Depression Scale (HADS). This instrument has been chosen because it is short, easy to use and interpret, translated to Arabic, valid, and reliable [31-34].

Previous studies tested the psychometric properties of the Arabic version of this instrument [31, 33, 34]. The Cronbach’s α reliability coefficients were .78 for anxiety subscale and .87 for the depression subscale indicating a very good internal consistency. Moreover, the anxiety subscale has a high sensitivity and the specificity at 86% and 87%, respectively [31, 33, 34]. Regarding the depression subscale these were 79% and 87%, respectively.

The total instrument consists of 14 items in two subscales; one for anxiety and one for depression. Each subscale consists of 7-items that were rated by the participants on a 0 to 3 scale, with 3 indicating higher symptom frequency and severity. The total score for each subscale can range from 0 to 21, with higher scores indicating higher levels of anxiety/depression.

Previous studies categorized the scores as the following: 0 to 7, normal; 8 to 10, mild; 11 to 14, moderate; and 15 to 21, severe anxiety/depression [31, 33, 34]. For the purposes of this study, participants were considered non-anxious/non-depressed if they have a score from 0-7, and anxious/depressed if they have a score from 8-21. To avoid categorizing participants who were experiencing transient anxiety/depression as persistently anxious/depressed, anxiety and depression were measured at the beginning of the semester for all students and at two-month follow-up. If the two measures (at the beginning of the semester and at two-month follow-up) were high (from 8-21), the participant was considered as persistently anxious/depressed. If the two measures (at the beginning of the semester and at two-month follow-up) were within normal (from 0-7), the participant was considered as persistently non (anxious/depressed). If one of the measurements was high (from 8-21) and the second was within normal (from 0-7), the participant was considered as transiently anxious/depressed.

#### 2.2.2 Absenteeism and GPA

The official semester at Jordanian universities according to academic calendar is composed of 16 weeks. However, the structure of the course is different between the undergraduate and graduate students. For a three credit hours course, the undergraduate students sit three (one-hour) sessions per week for 16 weeks resulting in 48 hours per semester. To meet the needs of the graduate students, the courses are structured differently. The student will sit one (three–hour) session per week for 16 weeks resulting in 48 hours per semester. Therefore, the total hours of absenteeism rate were divided by 48 then it was multiplied by 100% to get percentages; to avoid the discrepancy in the measurement of absenteeism rate.

To collect the data regarding the GPA, number of absenteeism and the gender of the students; the principal investigator checked the electronic system of the university. Course coordinators upload this information systemically into the system. These information are usually confirmed by the department head and the dean of the college on regular basis each semester which enhance the validity of the data. For the analysis purposes, the absenteeism percentage was divided into three categories as the following: 0-5%, 5.1-10%, more than 10%. The GPA is defined as the Grade Point Average of the semester when the study was conducted.

### 2.3 Procedure

The study was approved by the Institutional Review Board committee at Applied Science Private University. The principal investigator met with all students and explained the purpose of the study. Informed consent was obtained at enrollment if the student agreed to participate. Each student completed the HADS at the beginning of the semester and again after 2 months. These two times were chosen to avoid anxiety and depression resulting from examination.

### 2.4 Data Analysis

Data were analyzed using SPSS version 21.0. An alpha of .05 was set a priori. All research hypotheses were checked using ANOVA. When the main model was significant, a post hoc analysis was done using Least Significant Difference (LSD) test to determine which group differences were responsible for the significant main effect.

## 3 RESULTS

### 3.1 Sample Description

The sample consisted of 130 undergraduate students and 40 graduate students. Sixty percent of the sample (102 participants) was females. Forty-six participants were persistently depressed, 35 were transiently depressed, and 89 were persistently not depressed. Regarding anxiety, 40 participants were persistently anxious, 30 were transiently anxious, and 100 were persistently not anxious. Fifty eight percent of the sample has absenteeism rate between 0-5%, 26% of the sample has absenteeism rate between 5.1-10%, and 16% has absenteeism rate between greater than 10% (Table **1**). There were no differences in any of the characteristics among the three groups.

### 3.2 Hypotheses Testing: Hypothesis 1

There was a significant difference in the main model (F_(2,167)_ = 7.62, p <0.001). The post hoc analysis showed that the persistently anxious group has lower GPA than persistently non-anxious group (mean [SD], 64.1 [13.8] *vs*. 73.1 [12.3], P< .001). There were no other significant differences in the other comparisons. *Hypothesis 2:* There was a significant difference in the main model (F_(2,167)_ = 12.67, p <0.001). The post hoc analysis showed that the persistently anxious group has higher absenteeism rate than persistently non-anxious group (mean [SD], 7.62 [5.7] *vs*. 4.0 [3.4], P< .001) and higher than transiently anxious group also (mean [SD] 7.62 [5.7] *vs*. 4.7 [4.6], P< .05). There were no other significant differences in the other comparisons. *Hypothesis 3:* There was a significant difference in the main model (F_(2,167)_ = 7.95, p <0.001). The post hoc analysis showed that the persistently depressed group has lower GPA than persistently non-depressed group (mean [SD], 64.0 [13.8] *vs*. 73.2 [13.0], P< .001) and lower than transiently depressed (mean [SD], 64.0 [13.8] *vs*. 71.7 [10.6], P< .01). There were no other significant differences in the other comparisons. *Hypothesis 4:* There was a significant difference in the main model (F_(2,167)_ = 13.55, p <0.001). The post hoc analysis showed that the persistently depressed group has higher absenteeism rate than persistently non-depressed group (mean [SD], 7.52 [5.9] *vs*. 4.0 [3.7], P< .001) and higher than transiently depressed (mean [SD], 7.52 [5.9] *vs*. 3.4 [2.9], P< .001). There were no other significant differences in the other comparisons (Table **2**).

## 4 DISCUSSION

Results of this study showed that persistently anxious and depressed nursing students are more likely to have lower academic achievement as demonstrated by GPA and higher absenteeism rate than transiently non-anxious, non-depressed nursing students. There were no other significant differences in the other comparisons. This study responded to the gap in the literature investigating GPA and absenteeism in the context of anxiety and depression among nursing students at two different points of time. So, discussion of findings from this study will be in the light of other similar conclusions from other healthcare professions.

Several studies identified different levels of anxiety among nursing students at different stages of their collegial life. For example, some studies [28, 35, 36] shared the same conclusions that nursing students’ anxiety continues to increase as they go through the program to reach its highest level in their final year of the program. While, others found that nursing students' anxiety was at its highest level during the second year of the program [37]. These conclusions support the existence of different anxiety levels among nursing students along their collegial life. However, there was no linkage of anxiety to academic achievement or absenteeism in any of these studies.

The level of anxiety that nursing students experience can be explained by the progression through the developmental stages of the student [38]. The usual age of collegial life is 18-22 years old, which is the age when young people seek education and training to become successful adults [39]. This achievement is critical to successful adjustment and emotional well-being, while, failure may hinder emotional adjustment. Fear of failure provides a possible explanation for their anxiety [40]. Moreover, collegial age represents transition or bridge between adolescence and adulthood stages of life. This transition may hold some stress that can expose students for poor mental health which can affect their emotional well-being throughout the lifespan [41].

Persistent anxiety and depression are examples of these emotional consequences that may impair their progress in the nursing program [16, 26, 42]. To some extent, the hypotheses of this study, that persistently anxious and depressed students will have lower academic achievement is consistent with these findings as feelings of stress may be experienced during entrance to nursing school as a stage to transition to collegial life [43].

Investigating the literature, mixed results were found. Although no-nursing individuals were subjects of these studies, anxiety can affect learning and academic achievement was determined by overall level of mental health in most of the cases. For example, Yeh, *et al*. (2007) indicated that individuals with high levels of anxiety had poor academic achievement under stressful instruction and extreme levels of anxiety interferes with their attention [44]. A previous study also showed that extreme anxiety when facing an examination could impair individuals’ cognitive function and further impair their performance [45], which give further support to our findings from this study.

However, individuals with low levels of anxiety had better academic achievement under stressful instruction [46]. In one of the reviewed studies on medical student, learning under different levels of anxiety and depression, anxiety and depression follow up gave different directions of correlation (positive and negative) between levels of anxiety and academic achievement [46]. Another example, Yeh, *et al*. (2007) indicated that appropriate degrees of anxiety, especially concerning fear of failure, would self-reinforce the motivation system, and those with “fear of failure” had better performance in tough tasks than those with “hope of success”. Further studies are indicated to set a cutoff point or limits for the appropriate level of anxiety and to investigate the impact of this level among nursing students.

Depression and GPA are interrelated. As depression may impact GPA, in turn, lower GPA may result in Depression. Chen, *et al*., (2015) in their study of 700 Taiwanese nursing students, they found that depressive symptoms are significantly related to GPA, and, higher levels of depression were observed in students with low GPA, while those with higher GPA were less likely to report depressive symptoms [47]. Additional studies might be indicated to check the reverse impact of academic achievement on emotional wellbeing.

Regarding absenteeism, some studies failed to find a significant correlation between academic achievement and attendance [48-51]. However, others supported the hypothesis that absence from the class and the academic achievement are correlated. Absenteeism and not being physically available in the class can negatively impact the examination grades as demonstrated by GPA [52-56] and considerably lowers the students’ final grade (about 2 points in a 0-20-point grading scheme) [57]. In addition, some studies found that attendance was improved when it was tied to grades [58, 59] and a compulsory, though flexible, attendance policy contributes to improving students’ academic performance [60]. Further studies are indicated to investigate the impact of various attendance polices on absenteeism and GPA.

## CONCLUSION

Academic achievement is an important dimension for students. It is essential for educational institutions to assist students to develop emotionally as well as academically. This study provided an evidence for the impact of negative emotions such as anxiety and depression upon GPA and absenteeism. Moreover, this study showed that persistent anxiety and depression have a major effect on students' academic achievement and attendance to classes. It is highly recommended that administrators identify different sources of anxiety and depression and set strategies to control these negative emotions. Additionally, formal and periodic referral of students with early signs of anxiety and depressions to the psychosocial counselor should be planned for to detect and manage such symptoms.

## LIMITATIONS

This study was the first one in Jordan to include graduate student. However, the number of the graduate students that were included in this study was small. It is recommended to repeat this study with larger sample size of graduate students and to check the effect of interventions to control anxiety and depression on the outcomes of interest.

## Figures and Tables

**Table 1:** Sample characteristics based on the three levels of anxiety and depression (N=170).

**Characteristics**	**Persistently Anxious (n=40)**	**Transiently Anxious (n=30)**	**Non-Anxious (n=100)**
Age	26.2±7.6	25.8±6.9	26.9±7.1
Gender Male Female	17 (42.5) 23 (57.5)	14 (46.6) 16 (53.4)	37 (37) 63 (63)
Level of Education Graduate Undergraduate	9 (22.5) 31 (77.5)	7 (23.3) 23 (76.7)	24 (24) 76 (76)
**Characteristics**	**Persistently Depressed (n=46)**	**Transiently Depressed (n=35)**	**Non-depressed (n=89)**
Age	26.1±7.5	25.9±6.6	26.7±6.8
Gender Male Female	24 (52.1) 22 (47.9)	10 (28.6) 25 (71.4)	34 (38.2) 55 (61.8)
Level of Education Graduate Undergraduate	7 (15.2) 39 (84.8)	20 (57.1) 15 (42.9)	13 (14.6) 76 (85.4)

Values are Mean ± SD, or n (%).

**Table 2:** Post hoc analysis for hypotheses testing regarding GPA & Absenteeism rate.

**GPA**			
Level of Independent Variable	Compared With	Mean Difference	Sig
Persistently anxious	Non-anxious	-9.0	< .001
Persistently depressed	Non-depressed	-9.2	< .001
–	Transiently depressed	-7.7	< .01
**Absenteeism rate**			
**Level of Independent variable**	**Compared with**	**Mean difference**	**Sig**
Persistently anxious	Non-anxious	3.62	< .001
–	Transiently anxious	2.92	< .05
Persistently depressed	Non-depressed	3.52	< .001
–	Transiently depressed	4.12	< .001
